# CD206+ macrophage is an accelerator of endometriotic-like lesion via promoting angiogenesis in the endometriosis mouse model

**DOI:** 10.1038/s41598-020-79578-3

**Published:** 2021-01-13

**Authors:** Yosuke Ono, Osamu Yoshino, Takehiro Hiraoka, Erina Sato, Akiko Furue, Allah Nawaz, Hideki Hatta, Yoshiyuki Fukushi, Shinichiro Wada, Kazuyuki Tobe, Yasushi Hirota, Yutaka Osuga, Nobuya Unno, Shigeru Saito

**Affiliations:** 1 Department of Obstetrics and Gynecology, Teine Keijinkai Hospital, Sapporo, Japan; 2 Department of Obstetrics and Gynecology, Kitasato University School of Medicine, Sagamihara, Japan; 3 Department of Molecular and Medical Pharmacology, Faculty of Medicine, University of Toyama, Toyama, Japan; 4 Department of Diagnostic Pathology, Graduate School of Medicine and Pharmaceutical Sciences, University of Toyama, Toyama, Japan; 5 First Department of Internal Medicine, University of Toyama, Toyama, Japan; 6 Department of Obstetrics and Gynecology, The University of Tokyo, Tokyo, Japan; 7 Department of Obstetrics and Gynecology, University of Toyama, Toyama, Japan

**Keywords:** Endocrinology, Pathogenesis

## Abstract

In endometriosis, M2 MΦs are dominant in endometriotic lesions, but the actual role of M2 MΦ is unclear. CD206 positive (+) MΦ is classified in one of M2 type MΦs and are known to produce cytokines and chemokines. In the present study, we used CD206 diphtheria toxin receptor mice, which enable to deplete CD206+ cells with diphtheria toxin (DT) in an endometriosis mouse model. The depletion of CD206+ MΦ decreased the total weight of endometriotic-like lesions significantly (*p* < 0.05). In the endometriotic-like lesions in the DT group, a lower proliferation of endometriotic cells and the decrease of angiogenesis were observed. In the lesions, the mRNA levels of VEGFA and TGFβ1, angiogenic factors, in the DT group significantly decreased to approximately 50% and 30% of control, respectively. Immunohistochemical study revealed the expressions of VEGFA and an endothelial cell marker CD31 in lesions of the DT group, were dim compared to those in control. Also, the number of TGFβ1 expressing MΦ was significantly reduced compared to control. These data suggest that CD206+ MΦ promotes the formation of endometriotic-like lesions by inducing angiogenesis around the lesions.

## Introduction

Endometriosis is a gynecological disorder occurring in women of reproductive age^1–3^. The primary symptoms are severe pelvic pain and infertility^4,5^. Multiple lines of evidence suggest that inflammation plays a pivotal role for the pathogenesis of this disease and MΦs play central role for this process^1,6–8^.

It has been reported that the number of MΦs is elevated in the peritoneal fluid of women with endometriosis^6,8,10,11^, and a significant increase in MΦ numbers is also observed in the eutopic and ectopic endometrium in women with endometriosis^6,9,12^. Recent pieces of evidence suggest that MΦs directly affect endometriotic cells and promote the disease^6,13–15^. Activated MΦs in the peritoneal fluid and endometriotic implants produce numerous cytokines, such as including IL-1β, IL-6, IL-8, IL-12, and TNFα, which promote proliferation, angiogenesis, and inflammatory responses^1,16–22^. Using a rat model for endometriosis, Haber et al*.* demonstrated that peritoneal MΦ depletion with liposomal bisphosphonate resulted in a reduction in the development and growth of endometrium implants^23^. This finding suggests that MΦs play essential roles in the establishment of disease and its progression.

MΦs are known to be polarized by the distinct signals depending on their biological conditions^24–26^. MΦs have the plasticity and can be polarized into classically activated (M1) and alternatively activated (M2) MΦs. M2 MΦs are divided into M2a, M2b, M2c, and M2d subcategories. These MΦs differ in their cell surface markers, secreted cytokines and biological functions, suggesting that there are a variety of populations in MΦs^27–29^. In endometriosis, it has been reported that M2 MΦs are dominant in endometriotic lesions^13,14^. Bacci et al. showed that intraperitoneal injection of M2 MΦ promoted the growth of endometriotic like lesions in a mouse model, suggesting M2 MΦs are responsible for promoting endometriotic lesions^13^. However, the actual role of MΦ in the pathogenesis of endometriosis is unclear.

To elucidate the role of MΦ in vivo, the use of neutralization antibody has been utilized in eliminating MΦs but is less effective in removing MΦs within lesions owing to differential concentrations of antibody^30^. Therefore, in the present study, we used CD206 diphtheria toxin receptor (DTR) mice, which enable to deplete CD206+ MΦs, one of a specific marker of M2 MΦ, with diphtheria toxin (DT) injection^31,32^ to examine the role of CD206+ MΦ in progression of endometriosis.

## Material and methods

### Reagents and materials

Roswell Park Memorial Institute (RPMI)-1640 medium and Diphtheria Toxin (DT) were purchased from Sigma-Aldrich (St. Louis, MO, USA). Estradiol valerate was from Asuka Pharmaceutical Co., Osaka, Japan. Fetal bovine serum (FBS) was from Life Technologies (Tokyo, Japan). Antibiotics (a mixture of penicillin, streptomycin, and amphotericin B) were from Wako Pure Chemical Industries (Osaka, Japan).

### Immunohistochemistry

Paraffin-embedded tissues were cut 5-μm thick and mounted on slides. The sections of endometriotic-like lesions formed in endometriosis model mice were deparaffinized in xylene, rehydrated through a graded series of ethanol, and washed in water. Antigen retrieval was performed in 10 mM sodium citrate buffer (pH 6.0) by microwaving for 10 min and then cooling to room temperature. Control IgG was used as a negative control. Slide staining with antibodies was performed according to the manufacturer's instructions. The immunostaining was performed using specific antibodies to Ki-67 (Abcam, Tokyo, Japan, Cat# 15580, 1:100 dilution), Vascular endothelial growth factor (VEGFA) (Abcam, Tokyo, Japan, Cat# 46154, 1:100 dilution), CD206 (Abcam, Cat# 64693, 1:100 dilution), TGFβ1 (Abcam, Tokyo, Japan, Cat# 92486, 1:500 dilution), and CD31 (Abcam, Tokyo, Japan, Cat# 124432, 1:200 dilution). All images were taken with Keyence BZ-X800 (Keyence, Tokyo, Japan). Immunofluorescence analysis of endometriotic-like lesions was performed using CD206 and TGFβ1. The primary antibodies were incubated overnight at 4 °C, and as a second antibody, the gout anti-rabbit antibody (Alexa Fluor 488, ab150077, Abcam, Tokyo, Japan) was used. 4′,6-diamidino-2-phenylindole (DAPI; 1:500) was used to detect nuclei. Rabbit IgG was used instead of the primary antibody for negative control.

The intensity of the staining of VEGFA, CD31, and TGFβ1 was analyzed by a semi-quantitative method, H-scoring^33^. H-score was calculated by the following equation: H-score = ∑P_i_ × i, where i is the intensity of staining with a value of 0, 1, 2, or 3 (negative, weak, moderate, or strong, respectively) and P_i_ is the corresponding percentage of the cells. As for the Ki-67 evaluation, the percentage of Ki-67 positive cells per total cells was calculated.

### Model of endometriosis using CD206 DTR mouse

All animal experiments were approved by the ethical committee of University of Toyama (G2015MED-38 and A2013MED-33), and performed in accordance with animal experiment guidelines and regulations in University of Toyama. Female, CD206 DTR mice, from 12 to 20-weeks-old mice were used. We housed in a specific pathogen-free (SPF) animal facility with a controlled environment, 22–24 °C and 60–70% relative humidity, and on a light/dark cycle (12 h light/12 h dark) with food and water ad libitum. CD206 DTR mice are genetically engineered transgenic (Tg) mice based on the transgenic expression of the diphtheria toxin receptor (DTR) under the control of the CD206 promoter to ablate CD206+ MΦs^31,32,34^ specifically. In CD206 DTR mice, CD206+ MΦs are removed about more than 80% in various organs systemically, such as lung, adipose tissue, blood, spleen, and ovary^31,32,34^. Induction of endometriosis was studied as described previously^7^. Briefly, mice were injected s.c. with 100 μg/kg estradiol valerate in sesame oil once per week for three weeks. After three weeks, endometrium-rich fragments from donor mice were finely chopped using a razor blade. Fragments suspended in 0.6 ml phosphate buffered salts (PBS) were injected with an 18-gauge needle through the abdominal wall into the peritoneal cavity of recipient mice with the ratio of one donor to two recipients (designated day 0 when endometrial fragments were injected). We used wild type mice as donors and CD206 DTR mice as recipients. One week after endometrial inoculation, we checked the formation of endometriotic-like lesions in the peritoneal cavity by sacrificing a few mice. Then, the recipient mice underwent intraperitoneal injection of DT (DT group) or PBS for control group, every two days. DT of 20 ng/gram body weight was diluted with sterile PBS and injected intraperitoneally^31^. Two weeks after the inoculation of endometrial fragments, the recipient mice were sacrificed through cervical dislocation under anesthesia. Then, PBS (1 ml) was injected into the peritoneal cavity. After vigorous shaking, peritoneal fluid (PF) and PF cells were collected. Laparotomy was performed, and the numbers of endometriotic foci were counted. Each focus and uterus was excised to exclude as much normal surrounding tissues as possible. Immediately, the weight of the excised tissues was measured. During all the inspection procedures, examiners were blinded to the treatment given to each mouse.

### Reverse transcription (RT) and quantitative real-time polymerase chain reaction (PCR) analysis

Total RNA was extracted from mouse endometriotic-like lesions using the ISOGEN-II (NIPPON GENE, Tokyo, Japan) according to the manufacturer’s instructions. About 1 μg of total RNA was reverse-transcribed using Rever Tra Ace qPCR RT Master Mix with gDNA Remover (TOYOBO, Tokyo, Japan). For the quantification of various mRNA levels, real-time PCR was performed using the Mx3000P Real-Time PCR System (Agilent Technologies, CA, USA) according to the manufacturer’s instructions^31^. The PCR primers were selected from different exons of the corresponding genes to discriminate PCR products that might arise from possible chromosomal DNA contaminants. The SYBR Green thermal cycling conditions were one cycle of 95 °C for 30 s, and cycles of 95 °C for 10 s, 60 °C for 10 s and 72 °C 10 s). The primer sequences were as follows: Glyceraldehyde 3-phosphate dehydrogenase (GAPDH, NM_001289726.1: 801–822 and 883–862). CD206 (NM_008625.2: 3335–3355 and 3630–3611), TNFα (NM_013693.3: 435–455 and 556–533), IL-1β (NM_008361.4: 338–359 and 442–420), VEGFA (NM_001025257.3: 1103–1127 and 1261–1241), and TGFβ1(NM 1829–1810 and 1702–1722).　The relative mRNA levels were calculated using the standard curve method and were normalized to the mRNA levels of GAPDH^31^.

### Human peritoneal fluids MΦ (PF MΦ) and culture of human endometriotic stromal cells (ESCs)

The experimental procedures were approved by the institutional review board of Kitasato University (approved number: B18-265), and University of Toyama (Approved Number: 25–44) and signed informed consent for the use of samples was obtained from each patient. PFMΦs were purified as previously described^35^. Culture of primary ESCs was described elsewhere^7^. Human samples were taken and used under each patient’s consent. All experimental methods were carried out in accordance with relevant guidelines and regulations of Declaration of Helsinki.

### Statistical analysis

Data were evaluated by Mann Whitney using Jump version 10. *P* less than 0.05 was accepted as statistically significant.

## Results

### Endometriosis model using CD206 DTR mice

**Figure 1:**
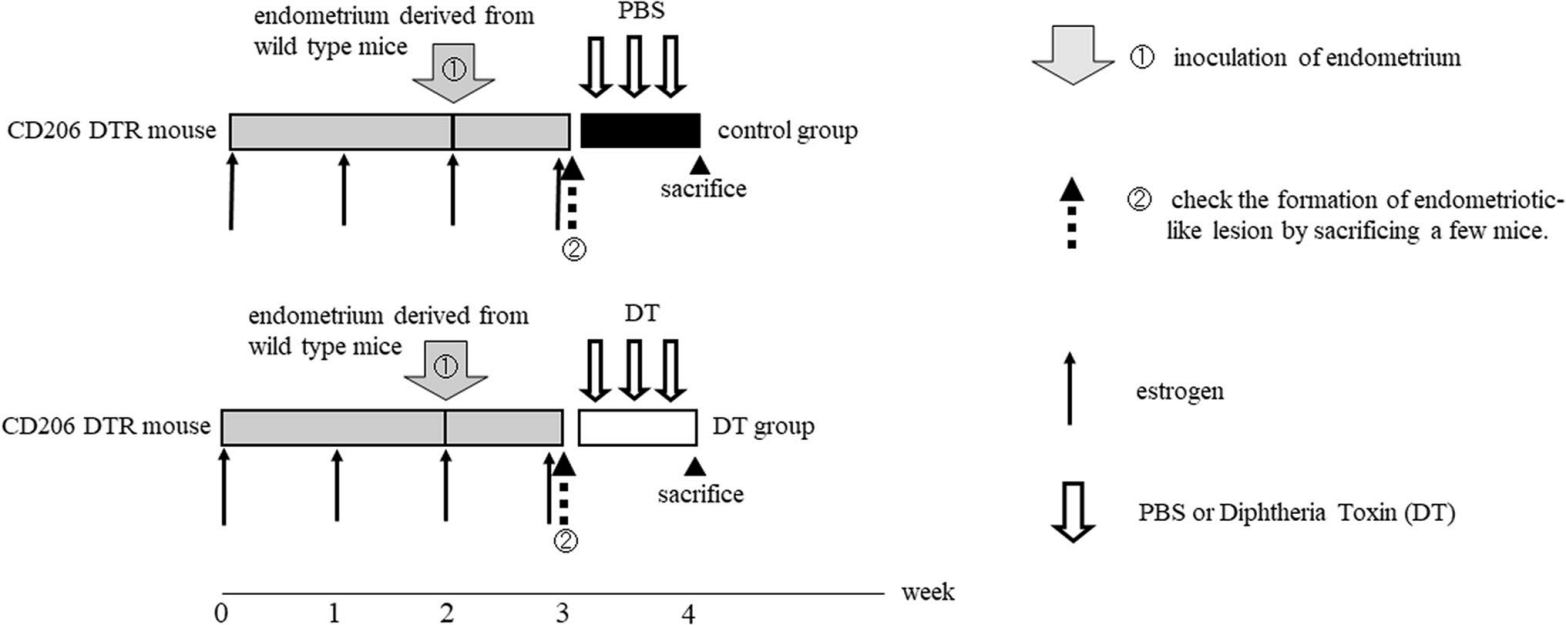
The depletion protocol of CD206+ macrophages (MΦ) in the CD206 DTR mouse-derived endometriosis model. After one week of inoculation of endometrial fragments derived from donor mice (①), the formation of an endometriotic-like lesion in the peritoneal cavity was confirmed in some mice. (②). The recipient mice underwent intraperitoneal injection of Diphtheria Toxin (DT) for DT group or phosphate buffered salts (PBS) for control group every two days starting from day 7 to day14 after endometrial inoculation. After 2 weeks of inoculation of the endometrium, the total weights of endometriotic-like lesions per mouse were investigated. Estrogen was injected every week.

**Figure 2:**
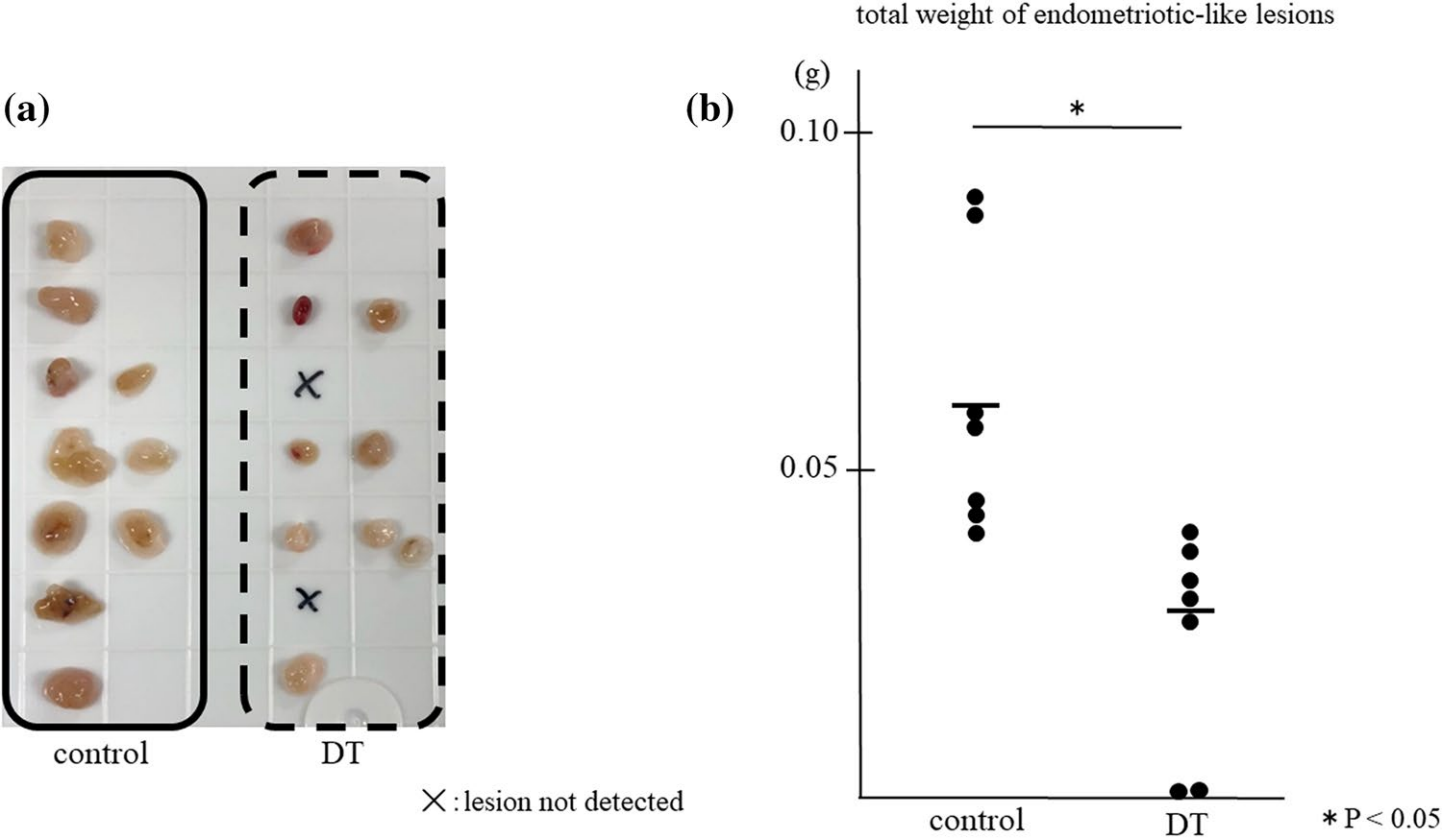
The depletion effect of CD206+ macrophages (MΦ) in the endometriosis mouse model. Representative appearances of endometriotic-like lesions are shown (**a**), and data of the total weight of lesions are shown as the dots and mean (**b**). In CD206 DTR mice, depletion of CD206+ MΦs was induced by injection of Diphtheria Toxin (DT).

As shown in Fig. 1, we induced endometriotic-like lesions using CD206 DTR mice. After one week of inoculation of endometrial fragments into the peritoneal cavity, we started to deplete CD206+ MΦs for a week and measured the total weight and the number of endometriotic-like lesions per mouse. As shown in Fig. 2, in CD206 DTR, the depletion treatment of CD206+ MΦ significantly decreased the total weight of endometriotic-like lesions (*p* < 0.05), but the number of lesions per mouse was not changed compared to control (1.4 ± 0.5 vs 1.2 ± 1.1).

We checked the depletion of CD206+ MΦ in peritoneal micro-environment by quantitative-PCR and found that in peritoneal fluid cells, about 80% of CD206+ MΦ were reduced in CD206DTR mice with DT administration (supplemental Fig. 1). The mRNA expression of CD11b, which is a pan MΦ marker, was not decreased significantly with DT stimuli, suggesting that the amount of total MΦs was not changed (Supplemental Fig. 1). As for other types of MΦs, the ratio of iNOS, a classical M1 MΦ marker, to CD11b was increased significantly, but the ratio of inflammatory MΦs producing TNFα to CD11b was not increased significantly (Supplemental Fig. 1).

### Depletion of CD206+ MΦ lead to a histological change of the endometriotic-like lesion

**Figure 3:**
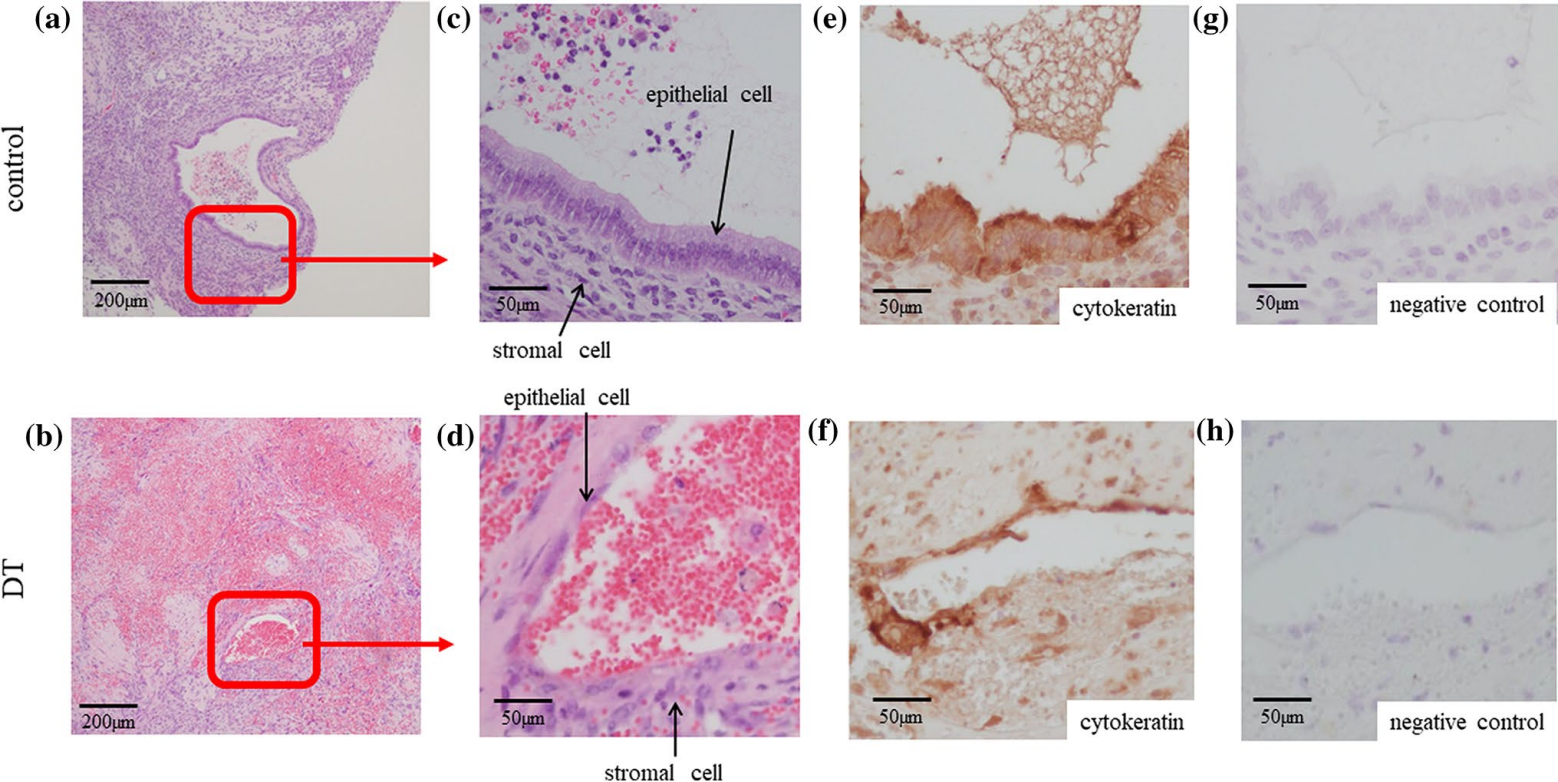
Representative microscopic picture of the endometriotic**-**like lesion of the DT and control group. In CD206 DTR mice, depletion of CD206+ macrophages (MΦ) was induced by injection of Diphtheria Toxin (DT). Representative endometriotic-like lesions in control (**a** and **c**) and DT group (**b** and **d**) were shown. The arrows indicate the endometriotic epithelial and stromal cells. Immunostaining of cytokeratin was performed in control (**a** and **c**) and DT (**b** and **d**) to confirm the presence of endometriotic epithelial cells in the same lesion as in both groups (**e**, **f**). Rabbit IgG was used for negative control (**g**, **h**).

Endometriotic lesion is composed by glandular epithelial cells and stromal cells. In the DT group, the appearance of endometriotic glandular epithelial cells was thinner, (Fig. 3b,d) compared to the appearance of epithelial cells in control (Fig. 3a,c). These surface cells covering lesions were confirmed to be epithelial cells by cytokeratin staining (Fig. 3e,f). Figure 3g,h show the negative control of Fig. 3e,f, respectively.

**Figure 4:**
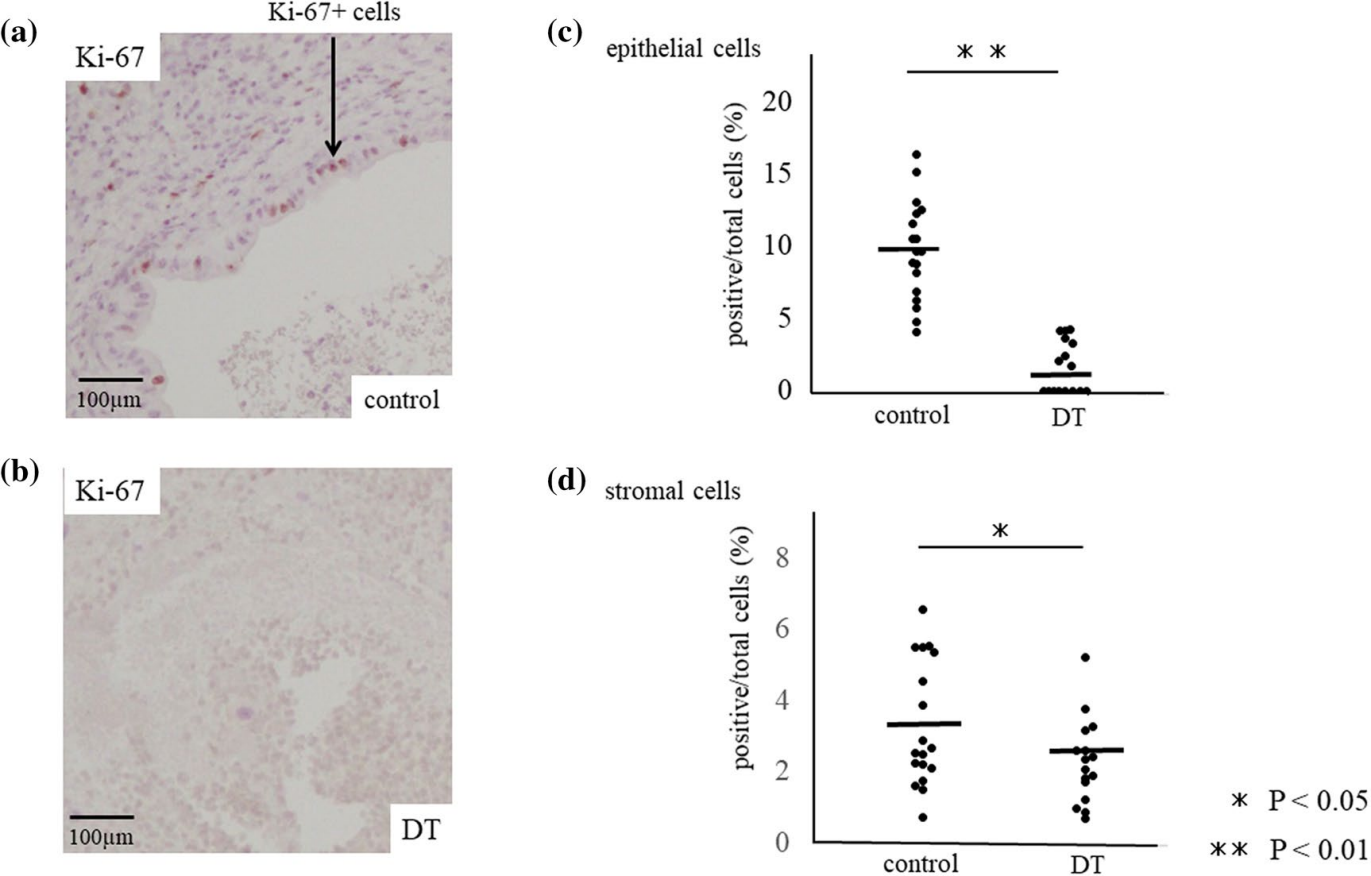
Cell proliferation at the endometriotic-like lesion of DT and control group. Immuno-staining of Ki-67, a proliferation marker, was examined in control (**a**) and DT (**b**) group, and the percentage of Ki-67 positive cells per total cells was calculated in glandular epithelial cells and stromal cells, respectively (**c** and **d**). The arrow indicates the endometriotic epithelial cells. Data were evaluated by Mann Whitney U. *P* less than 0.05 was accepted as statistically significant.

The positive ratio per total cells of proliferation marker, Ki-67 + cells, was significantly decreased in both the glandular epithelial cells and stromal cells of endometriotic-like lesions of DT group, receptively (1.6 ± 1.8 and 2.3 ± 1.2%, mean ± SD) compared to control (9.7 ± 13.4% and 3.3 ± 1.8, *p* < 0.001) (Fig. 4a–d), suggesting that lower proliferation ratio of endometriotic-like cells resulted in a decrease of endometriotic-like lesions in the absence of CD206+ MΦ.

### The possible mechanism of CD206+ MΦ in the progression of endometriotic lesions

**Figure 5:**
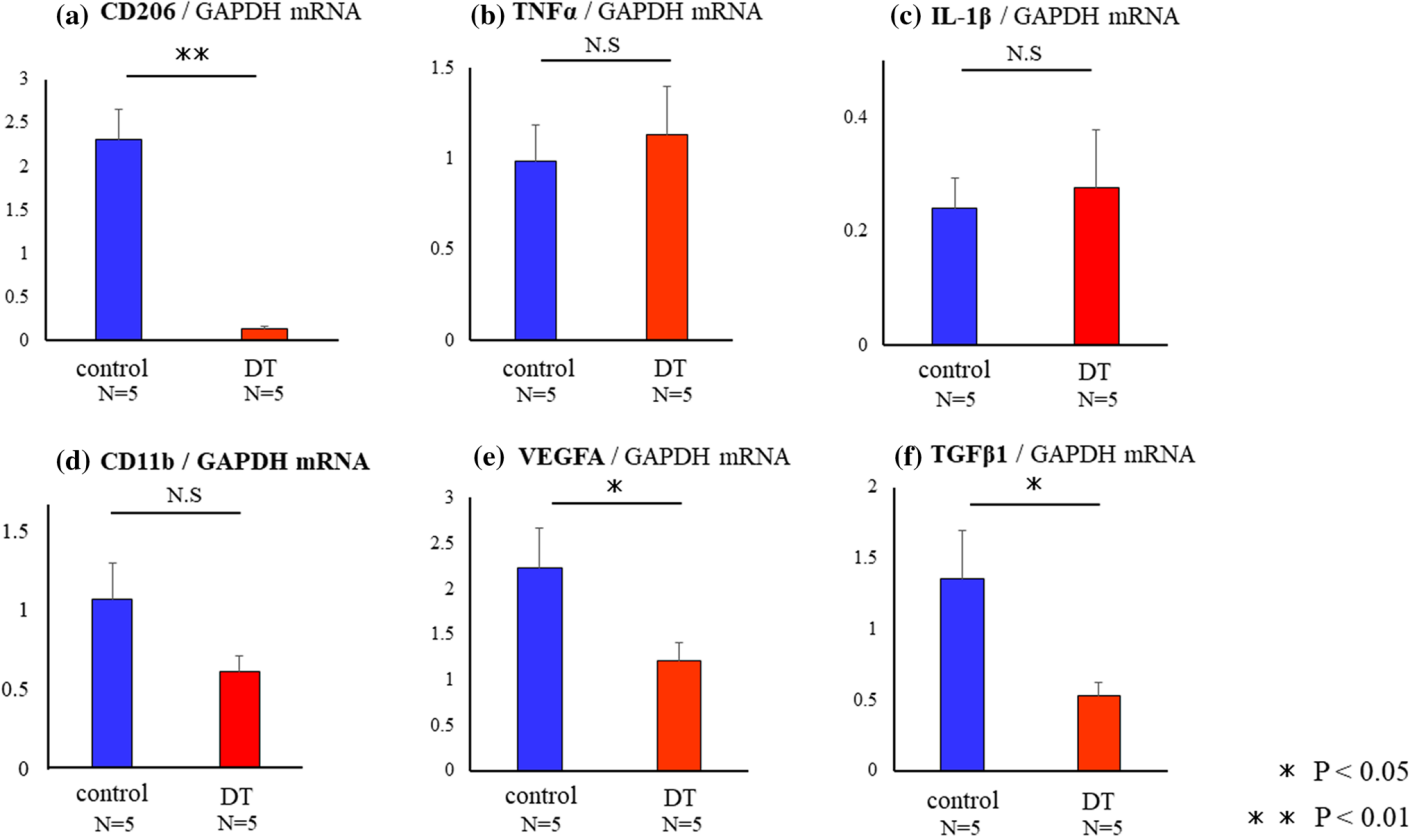
The expressions of mRNA in endometriotic-like lesions in control and DT injected mice. In CD206 DTR mice, depletion of CD206+ macrophages (MΦ) were induced by injection of Diphtheria Toxin (DT). The expression of CD206 (**a**), TNFα (**b**), IL-1β (**c**), CD11b (**d**), VEGFA (**e**), and TGFβ1 (**f**) mRNA expressions were measured with quantitative PCR in control and DT group. Data were normalized by GAPDH mRNA levels to show the relative abundance. Representative data from three different experiments were shown as the mean ± SEM. *p < 0.05.

To investigate the reducing mechanism of endometriotic-like lesions by the depletion of CD206+ MΦ, we checked the critical factors for the formation of endometriotic-like lesions at the mRNA level. As shown in Fig. 5a, the depletion of CD206+ MΦ significantly decreased the expression of CD206 mRNA by more than 90% compared to control (*p* < 0.01) in the lesions. Although the levels of TNFα and IL-1β were comparable in control and DT group (,c), the mRNA expression of CD11b, a pan- MΦ marker, was not changed (Fig. 5d). The proportion of CD206/CD11b was significantly decreased in DT group, while that of TNFα/CD11b was not changed in both groups (data not shown). The expression of VEGFA (Fig. 5e) and TGFβ1 (Fig. 5f) in DT group were significantly decreased to the levels of approximately 50% and 30% of control, respectively (*p* < 0.05, *p* < 0.05).

### CD206+ MΦ contributes to the angiogenesis of endometriotic-like lesions.

**Figure 6:**
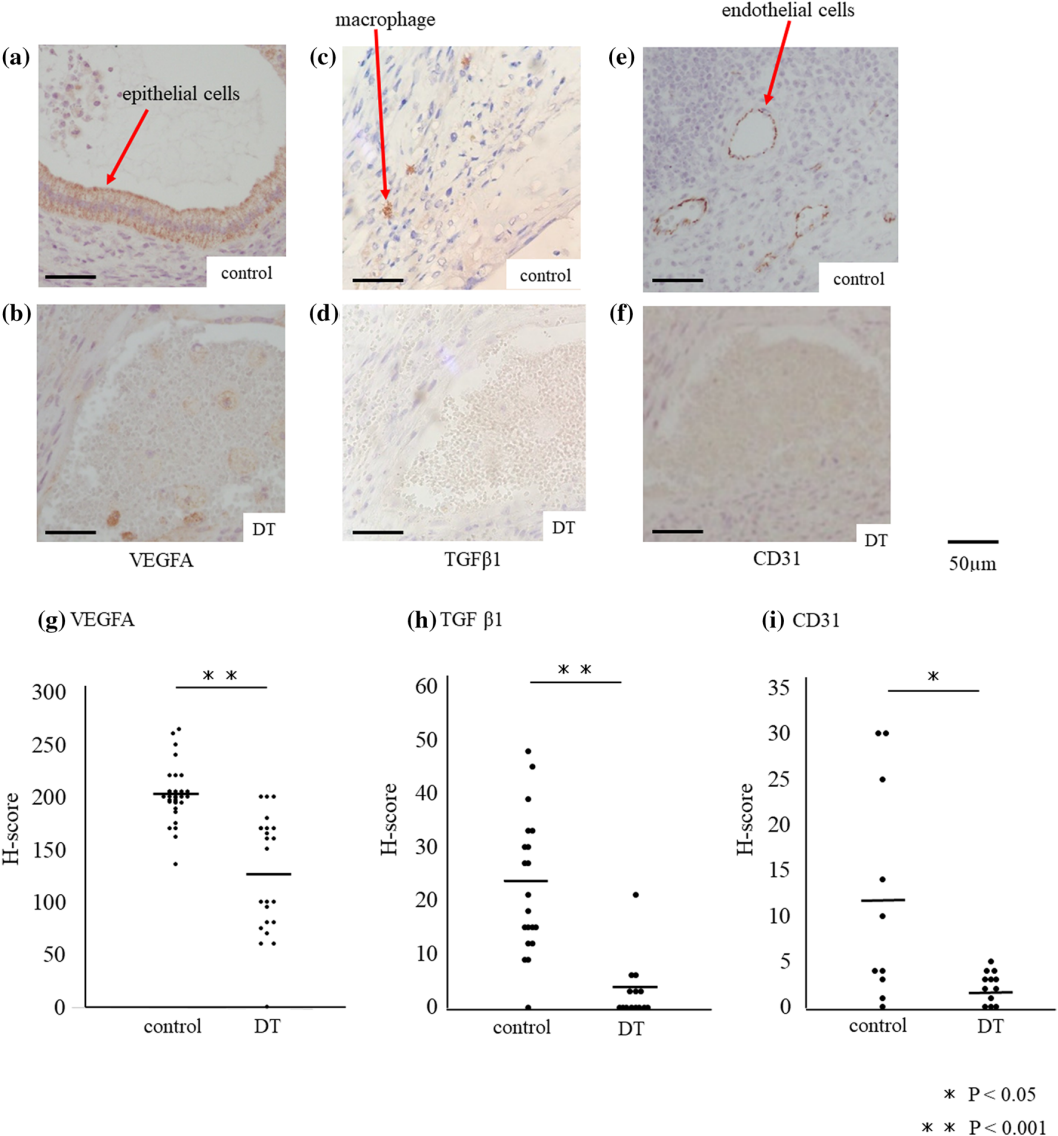
The expressions of VEGF, TGF-β and CD31 protein in endometriotic-like lesions. In CD206 DTR mice, depletion of CD206+ macrophages (MΦ) were induced by injection of Diphtheria Toxin (DT). The immunohistochemistry of VEGF (**a** and **b**), TGFβ1 (**c** and **d**), and a marker of the endothelium, CD31 (**e** and **f**), were examined in the endometriotic like lesions of control mice (control) and CD206+ MΦ depletion mice (DT). H-scores of VEGFA (**g**), TGFβ1 (**h**), and CD31 (**i**) immunostaining in endometriotic-like lesions were calculated and compared in control and DT mice. Data were evaluated by Mann Whitney U.* P* less than 0.05 was accepted as statistically significant.

**Figure 7:**
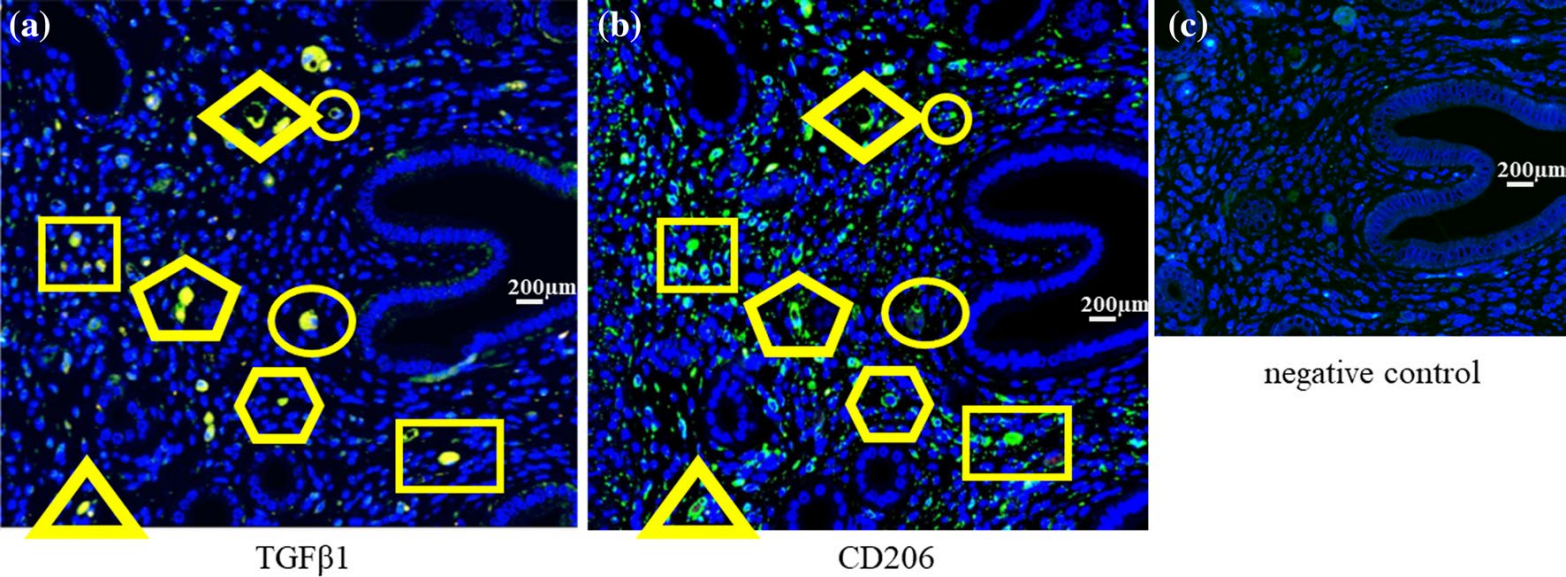
The expression of TGFβ and CD206 in endometriotic-like lesions. Using serial sections of endometriotic-like lesions induced in wild mice, antibodies of anti-TGFβ (**a**) or CD206 (**b**), a marker of macrophage (MΦ), were used. As a second antibody, the gout anti-rabbit antibody was used. 4′,6-diamidino-2-phenylindole (DAPI) was used to detect nuclei. In the serial section, each cell marked in the same shape was identical. Rabbit IgG was used instead of the primary antibody for negative control (**c**).

We investigated the localization of VEGFA and TGFβ1 protein in the lesions. Immunohistochemical study revealed, in the DT group, the expression of VEGFA in especially epithelial cells, was significantly lower (*p* < 0.001) compared to control (Fig. 6a,b,g H-score; 201.8 ± 26.7 vs 126.7 ± 56.0, *p* < 0.001). Also, the number of TGFβ1-expressing cells was significantly reduced compared to control (Fig. 6c,d,h; H-score; 22.7 ± 12.9 vs 3.0 ± 5.6, *p* < 0.001). Immunofluorescence study showed that TGFβ1 staining cells are CD206 positive cells, indicating that CD206+ MΦs are the main cells that produce TGFβ1 (Fig. 7). The expression of CD31, an endothelial cell marker, was significantly decreased in the endometriotic-like lesions with the depletion of CD206+ MΦ compared to control (*p* < 0.05, Fig. 6e,f,i, H-score; 12.1 ± 12.0 vs 2.2 ± 1.7, *p* < 0.05).

## Discussion

In endometriosis, it has been reported that M2 MΦs are dominant in endometriotic lesions^13,14^, but the roles of MΦ for the pathogenesis of endometriosis are remained to be clarified. To understand the dynamics and roles of MΦ in the pathogenesis of endometriosis, animal models might be a useful tool. Recently, Johan et al. investigated whether infiltrating MΦs acquire functionally different phenotypes, M1 or M2 MΦ, during lesion development in an endometriosis mice model^36^. They showed that until day 7, after grafting endometrial tissues to mice, M1 MΦs are dominant, while M2 MΦs exhibit dominancy after day 7^36^. Therefore, in the endometriosis mouse model, M1 to M2 MΦ shift occurs on day 7 after the inoculation of endometrial tissue. This is consistent with a transition from classical M1 MΦ activity to an alternate M2 profile, which correlates to the findings of initially acute inflammation followed by tissue remodeling in the process of development of endometirosis^36^.

Given that some M2 MΦs have a role of suppressing inflammation^37^, the contribution of M2 MΦ to the endometriosis-like lesions is uncertain, suppressive, or progressive. To address this question, we used CD206 DTR mice and depleted CD206+ MΦs exclusively in mice from day 7 after induction of endometriotic-like lesions, when the shift from M1 to M2 MΦ could occur in endometriotic lesions^36^. CD206 is known to be a type 1 mannose receptor and one of M2a subtype markers in M2 MΦs. These MΦs secrete cytokines such as TGFβ1 and IL-10 and chemokines such as CCLs^37^. In CD206 DTR mice model, CD206+ MΦs were depleted by more than 80% in endometriotic-like lesion and PF cells (Fig. 5a and Supplemental Fig. 1). At mRNA levels, although the ratio of iNOS/CD11b was increased in PF cells, the mRNA expression of CD11b was unchanged, and there was no increase of TNFα/CD11b, suggesting that inflammatory MΦs which exacerbate endometriotic lesions^6,38^ did not increase in both PF cells and lesions. We found that the depletion of CD206+ MΦ resulted in the decrease of the size of endometriotic-like lesions without affecting the number of lesions, implying that the role of CD206+ MΦ is an accelerator of endometriotic-like disease in mice model. Duan et al. used CD11b DTR mice in which pan MΦs could be depleted with DT treatment, and showed the reduction of lesion weight in an endometriosis mice model. Moreover, they also presented that the adoptive transfer of M2a, a subtype of M2 MΦ, systemically after the MΦ depletion significantly increased the weight of endometriotic lesions^39^, suggesting that M2a MΦ play a role in the progression of the endometriotic lesion. In consistent with the notion, as CD206 is one of M2a subtype markers in M2 MΦs^37^, our present results also suggest that CD206+ MΦs are involved in the exacerbation of endometriosis. In the DT group, endometriotic lesions exhibited thinner appearance, and lower proliferation rate of epithelial and stromal cells compared to control (Fig. 4), suggesting that CD206+ MΦs influence the growth of endometriotic cells. Among endometriosis-related factors, in the DT group, the levels of TGFβ1 and VEGFA mRNA, known as angiogenic factors^40,41^, were decreased in endometriotic-like lesions. In accordance with the notion, CD31, an endothelial cell marker, was almost disappeared in the endometriotic-like lesions with the depletion of CD206+ MΦ. It has been reported that the administration of bevacizumab, a VEGF-A antibody, resulted in reduced lesion formation in mouse endometriosis model studies^41^, suggesting neovascularization of ectopic endometrial tissue is crucial in the development of the endometriotic-like lesion.

In the present study, immunohistochemical analysis showed that CD206+ MΦs expressed TGFβ1 (Fig. 7) and produced high level of VEGFA in the endometriotic-like lesions (Fig. 6). Therefore, we performed an in vitro study to examine these relationships. It is well known that IL-33, an alarmin, deviates macrophage to classical M2 type^42–45^. We also have reported that IL-33 induced CD206+ MΦs in human peritoneal MΦs^35^. In the present study, we found that induced CD206+ MΦs with recombinant IL-33 stimuli (100 ng/ml, 8hrs) increased the expression of TGFβ1 mRNA (Supplemental Fig. 2). In addition, to investigate the relationship between TGFβ1 derived from CD206+ MΦ and VEGFA of the endometriotic lesion, we checked the mRNA expression of VEGFA in human endometriotic stromal cells (ESCs) with TGFβ1 stimulation and found the significant increase of VEGFA mRNA expression (Supplemental Fig. 2) suggesting that increased expression of TGFβ1 in CD206-skewed-MΦ may induce angiogenesis via increasing VEGFA expression in ESCs. TGFβ1 is related to angiogenesis^40^ and is also reported to induce VEGFA in other cells^46^. Therefore, the reduction in TGFβ1 expression by the depletion of CD206+ MΦ might have led to the decrease in angiogenesis, resulting in the reduction of endometriotic-like lesion.

In endometriosis patients, it is reported that Natural Killer (NK) cells impair their function in the intra-peritoneal environment, which makes endometriotic cells more likely to survive and proliferate in the abdominal cavity. TGFβ1 is known to be increased in the peritoneal fluids of patients with endometriosis^47^ and to exacerbate endometriosis by decreasing NK cell activity^48^. Therefore, decreasing of TGFβ1 by the depletion of CD206+ MΦ may contribute to the recovery of NK activity and result in the reduction of endometriosis lesions. Further studies are necessary to clary the role of CD206+ MΦ in the pathogenesis of endometriosis.

In the present study, we proved that CD206+ MΦ played a vital role in the promotion of endometriosis via inducing angiogenesis using CD206 DTR mice. Therefore, CD206+ MΦ might be a target for a new therapy for endometriosis. One can speculate that the depletion of CD206+ MΦ using a specific antibody could be a strategy. Further studies are needed to prove these notions.

## Supplementary Information

Supplementary Figures.

## References

[1] Zondervan KT (2018). Endometriosis. . Nat. Rev. Dis. Prim..

[2] Eskenazi B, Warner ML (1997). Epidemiology of endometriosis. Obstet. Gynecol. Clin. North Am..

[3] Shafrir AL (2018). Risk for and consequences of endometriosis: a critical epidemiologic review. Best Pract. Res. Clin. Obstet. Gynaecol..

[4] Ziegler DD, Borghese B, Chapron C (2010). Endometriosis and infertility: pathophysiology and. Lancet.

[5] Practice T, Medicine R (2012). Endometriosis and infertility: a committee opinion. Fertil. Steril..

[6] Capobianco A (2013). Endometriosis, a disease of the macrophage. Front. Immunol..

[7] Yamada-Nomoto K (2016). Alpha-7 nicotinic acetylcholine receptor (nAChR) agonist inhibits the development of endometriosis by regulating inflammation. Am. J. Reprod. Immunol..

[8] Izumi G (2018). Involvement of immune cells in the pathogenesis of endometriosis. J. Obstet. Gynaecol. Res..

[9] Berbic M (2009). Macrophage expression in endometrium of women with and without endometriosis. Hum. Reprod..

[10] Halme J, Becker S, Haskill S (1987). Altered maturation and function of peritoneal macrophages: possible role in pathogenesis of endometriosis. Am. J. Obstet. Gynecol..

[11] Hill JA, Faris HM, Schiff I, Anderson DJ (1988). Characterization of leukocyte subpopulations in the peritoneal fluid of women with endometriosis. Fertil. Steril..

[12] Hogg C, Horne AW, Greaves E (2020). Endometriosis-associated macrophages: origin, phenotype, and function. Frontiers Endocrinol..

[13] Bacci M (2009). Macrophages are alternatively activated in patients with endometriosis and required for growth and vascularization of lesions in a mouse model of disease. Am. J. Pathol..

[14] Itoh F (2013). Possible involvement of signal transducer and activator of transcription-3 in cell–cell interactions of peritoneal macrophages and endometrial stromal cells in human endometriosis. Fertil. Steril..

[15] Shao J (2016). Macrophages promote the growth and invasion of endometrial stromal cells by downregulating IL-24 in endometriosis. Reproduction.

[16] Conti HR, Gaffen SL (2010). Host responses to Candida albicans: Th17 cells and mucosal candidiasis. Microbes Infect..

[17] Yoshino O (2004). Possible pathophysiological roles of mitogen-activated protein kinases (MAPKs) in endometriosis. Am. J. Reprod. Immunol..

[18] Osuga Y (2011). Lymphocytes in endometriosis. Am. J. Reprod. Immunol..

[19] Ziętek A, Futyma K, Nowakowski Ł, Gogacz M, Rechberger T (2015). Progress on macrophage’s proinflammatory products as markers of acute endometriosis. J. Acute Dis..

[20] Kyama CM (2009). Role of cytokines in the endometrial-peritoneal cross-talk and development of endometriosis. Frontiers in Bioscience Elite.

[21] Laganà AS (2019). The pathogenesis of endometriosis: molecular and cell biology insights. Int. J. Mol. Sci..

[22] Riccio LGC (2018). Immunology of endometriosis. Best Pract. Res. Clin. Obstet. Gynaecol..

[23] Haber E (2009). Peritoneal macrophage depletion by liposomal bisphosphonate attenuates endometriosis in the rat model. Hum. Reprod..

[24] Mantovani A (2004). The chemokine system in diverse forms of macrophage activation and polarization. Trends Immunol..

[25] Canton J (2014). Phagosome maturation in polarized macrophages. J. Leukoc. Biol..

[26] Chistiakov DA (2015). Macrophage phenotypic plasticity in atherosclerosis: The associated features and the peculiarities of the expression of inflammatory genes. Int. J. Cardiol..

[27] Roszer T (2015). Understanding the mysterious M2 macrophage through activation markers and effector mechanisms. Mediators Inflamm..

[28] Qian BZ, Pollard JW (2010). Macrophage diversity enhances tumor progression and metastasis. Cell.

[29] Chávez-Galán L, Olleros ML, Vesin D, Garcia I (2015). Much more than M1 and M2 macrophages, there are also CD169+ and TCR+ macrophages. Front. Immunol..

[30] van Rooijen and Annemarie Sanders, N. & Sanders, A. Elimination, blocking, and activation of macrophages: three of a kind? *J. Leukoc. Biol.* **62**, 702–709 (1997). 10.1002/jlb.62.6.702 9400810

[31] Ono Y (2018). CD11c+ M1-like macrophages (MΦs) but not CD206+ M2-like MΦ are involved in folliculogenesis in mice ovary. Sci. Rep..

[32] Nawaz A (2017). CD206+ M2-like macrophages regulate systemic glucose metabolism by inhibiting proliferation of adipocyte progenitors. Nat. Commun..

[33] McCarty KS, Miller LS, Cox EB, Konrath J (1985). Estrogen receptor analyses. Correlation of biochemical and immunohistochemical methods using monoclonal antireceptor antibodies. Arch. Pathol. Lab. Med..

[34] Kambara K (2015). In vivo depletion of CD206+ M2 macrophages exaggerates lung injury in endotoxemic mice. Am. J. Pathol..

[35] Ono Y (2020). IL-33 Exacerbates endometriotic lesions via polarizing peritoneal macrophages to M2 subtype. Reprod. Sci..

[36] Johan MZ, Ingman WV, Robertson SA, Hull ML (2019). Macrophages infiltrating endometriosis-like lesions exhibit progressive phenotype changes in a heterologous mouse model. J. Reprod. Immunol..

[37] Yao Y, Xu XH, Jin L (2019). Macrophage polarization in physiological and pathological pregnancy. Frontiers Immunol..

[38] Agic A (2006). Is endometriosis associated with systemic subclinical inflammation?. Gynecol. Obstet. Invest..

[39] Duan J, Liu X, Wang H, Guo S-W (2018). The M2a macrophage subset may be critically involved in the fibrogenesis of endometriosis in mice. Reprod. Biomed. Online.

[40] Madri JA, Pratt BM, Tucker AM (1988). Phenotypic modulation of endothelial cells by transforming growth factor-beta depends upon the composition and organization of the extracellular matrix. J. Cell Biol..

[41] Ricci AG, Olivares CN, Bilotas MA, Meresman GF, Barañao RI (2011). Effect of vascular endothelial growth factor inhibition on endometrial implant development in a murine model of endometriosis. Reprod. Sci..

[42] Hazlett LD (2010). IL-33 shifts macrophage polarization, promoting resistance against Pseudomonas aeruginosa keratitis. Investig. Ophthalmol. Vis. Sci..

[43] Tu L (2017). IL-33-induced alternatively activated macrophage attenuates the development of TNBS-induced colitis. Oncotarget.

[44] Kurowska-Stolarska M (2009). IL-33 Amplifies the polarization of alternatively activated macrophages that contribute to airway inflammation. J. Immunol..

[45] Lott JM, Sumpter TL, Turnquist HR (2015). New dog and new tricks: evolving roles for IL-33 in type 2 immunity. J. Leukoc. Biol..

[46] Kaminska B, Wesolowska A, Danilkiewicz M (2005). TGF beta signalling and its role in tumour pathogenesis. Acta Biochim. Pol..

[47] Oosterlynck DJ, Meuleman C, Waer M, Koninckx PR (1994). Transforming growth factor-beta activity is increased in peritoneal fluid from women with endometriosis - PubMed. Obs. Gynecol..

[48] Jeung IC, Cheon K, Kim MR (2016). Decreased cytotoxicity of peripheral and peritoneal natural killer cell in endometriosis. BioMed Res. Int..

