# Supplementary material for: CD206+ macrophage is an accelerator of endometriotic-like lesion via promoting angiogenesis in the endometriosis mouse model
Source: Sci Rep. 2021 Jan 13;11:853. doi: 10.1038/s41598-020-79578-3 (PMC7807007; doi:10.1038/s41598-020-79578-3)
Supplement: Supplementary file 1 — Supplementary Figures. [file 41598_2020_79578_MOESM1_ESM.docx]

**Supplemental data**

**CD206+ Macrophage is an accelerator of endometriotic-like lesion via promoting angiogenesis in the endometriosis mouse model**

Yosuke Ono^1)^, Osamu Yoshino^2)^, Takehiro Hiraoka^2)^, Erina Sato^2)^, Akiko Furue^2)^ Allah Nawaz^3)^, Hideki Hatta^4)^, Yoshiyuki Fukushi^1)^, Shinichiro Wada^1)^, Kazuyuki Tobe^5)^, Yasushi Hirota^6)^, Yutaka Osuga^6)^, Nobuya Unno^2)^and Shigeru Saito^7)^

1. Department of Obstetrics and Gynecology, Teine Keijinkai Hospital
2. Department of Obstetrics and Gynecology, Kitasato University School of Medicine
3. Department of Molecular and Medical Pharmacology, Faculty of Medicine, University of Toyama
4. Department of Diagnostic Pathology, Graduate School of Medicine and Pharmaceutical Sciences, University of Toyama
5. First Department of Internal Medicine, University of Toyama
6. Department of Obstetrics and Gynecology, The University of Tokyo
7. Department of Obstetrics and Gynecology, University of Toyama


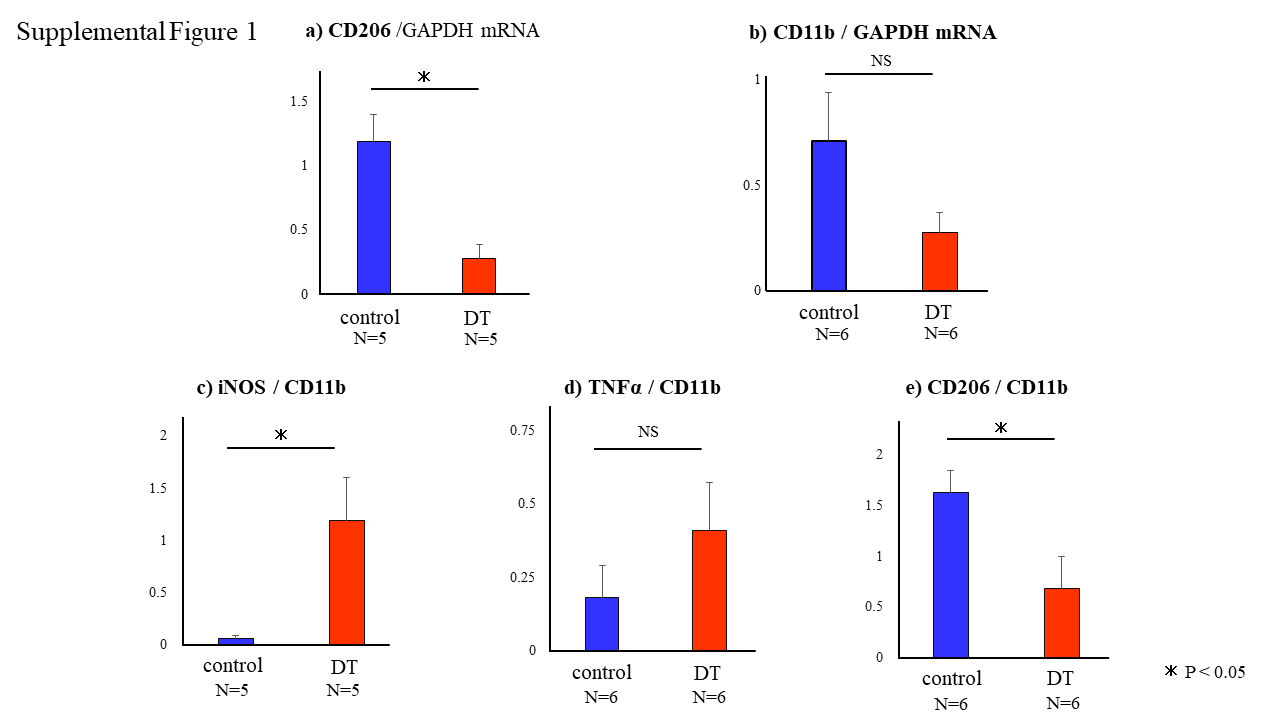


**Supplemental Figure 1. The** **expressions of mRNA in peritoneal fluid (PF) cells in control and DT group.**

In CD206 DTR mice, depletion of CD206+ macrophages (MΦ) were induced by injection of Diphtheria Toxin (DT). PF cells were collected by washing peritoneal cavity, and the mRNA expression of CD206 (a) and CD11b (b) were measured with quantitative PCR in control and DT group. CD11b, iNOS and TNFα were used as a total, a classical M1 type and a pro-inflammatory macrophage marker, respectively. The expression ratios of iNOS /CD11b, TNFα/CD11b, and CD206/CD11b in mRNA level were shown in (c), (d), (e), in control and DT group, respectively. Data of relative abundance were shown as the mean ± SEM. *: P<0.05.


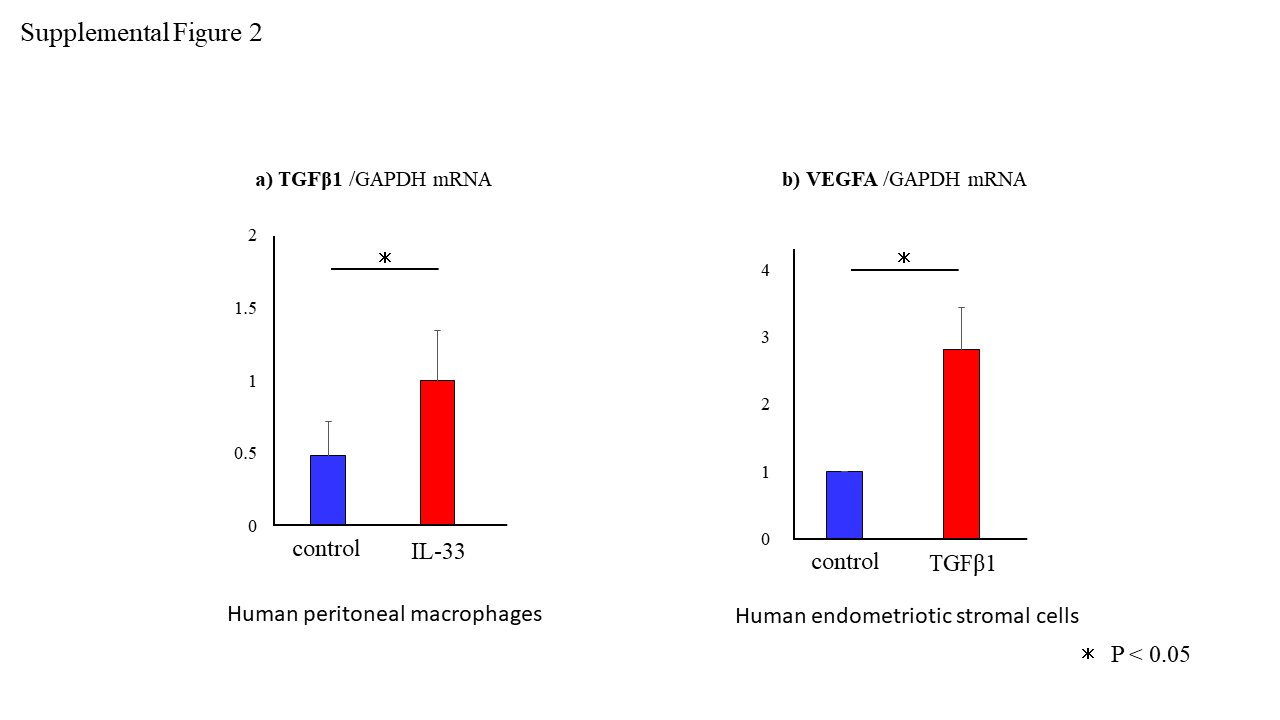


**Supplemental Figure 2. The mRNA expression of TGFβ1 and VEGFA.**

Human peritoneal macrophages without endometriosis were stimulated by IL-33

(100 ng/ml, 8 hrs). MΦs were purified as previously described ^35^. The mRNA

expression of TGFβ1 was examined by quantitative-PCR and shown in a). The

mRNA expression of VEGF in human endometriotic stromal cells (ESCs) with TGFβ1 stimuli was shown in b). Culture of primary ESCs was described elsewehere^7^. Data were normalized by GAPDH mRNA levels to show the relative abundance. Representative data from three different experiments were shown as the mean ± SEM. *: P<0.05.
